# Tumor Suppressor MicroRNA-27a in Colorectal Carcinogenesis and Progression by Targeting SGPP1 and Smad2

**DOI:** 10.1371/journal.pone.0105991

**Published:** 2014-08-28

**Authors:** Yonghua Bao, Zhiguo Chen, Yongchen Guo, Yansheng Feng, Zexin Li, Wenliang Han, Jianguo Wang, Weixing Zhao, Yunjuan Jiao, Kai Li, Qian Wang, Jiaqi Wang, Huijuan Zhang, Liang Wang, Wancai Yang

**Affiliations:** 1 Department of Immunology, Xinxiang Medical University, Xinxiang, China; 2 Department of Pathology, Xinxiang Medical University, Xinxiang, China; 3 Department of Laboratory Medicine, Xinxiang Medical University, Xinxiang, China; 4 Department of Pathophysiology, Xinxiang Medical University, Xinxiang, China; 5 Department of Surgery, the First Affiliated Hospital, Xinxiang Medical University, Weihui, China; 6 Department of Gastroenterology, Xinxiang Central Hospital, Xinxiang Medical University, Xinxiang, China; 7 Department of Pathology, Medical College of Wisconsin, Milwaukee, Wisconsin, United States of America; 8 Department of Pathology, University of Illinois at Chicago, Chicago, Illinois, United States of America; University of Barcelona, Spain

## Abstract

The aberrant expression of microRNAs (miRNAs) is associated with colorectal carcinogenesis, but the underlying mechanisms are not clear. This study showed that the miRNA-27a (miR-27a) was significantly reduced in colorectal cancer tissues and colorectal cancer cell lines, and that the reduced miR-27a was associated with distant metastasis and colorectal cancer clinical pathological stages–miR-27a was lower at stages III/IV than that at stage II. Bioinformatic and systemic biological analysis predicted several targets of miR-27a, among them SGPP1 and Smad2 were significantly affected. SGPP1 and Smad2 at mRNA and protein levels were negatively correlated with miR-27a in human colorectal cancer tissues and cancer cell lines. Increased miR-27a significantly repressed SGPP1 and Smad2 at transcriptional and translational levels. Functional studies showed that increasing miR-27a inhibited colon cancer cell proliferation, promoted apoptosis and attenuated cell migration, which were also linked to downregulation of p-STAT3 and upregulation of cleaved caspase 3. *In vivo*, miR-27a inhibited colon cancer cell growth in tumor-bearing mice. Taken together, this study has revealed miR-27a as a tumor suppressor and has identified SGPP1 and Smad2 as novel targets of miR-27a, linking to STAT3 for regulating cancer cell proliferation, apoptosis and migration in colorectal cancer. Therefore, miR-27a could be a useful biomarker for monitoring colorectal cancer development and progression, and also could have a therapeutic potential by targeting SGPP1, Smad2 and STAT3 for colorectal cancer therapy.

## Introduction

Colorectal cancer is one of the most common malignant diseases worldwide, but the causes of colorectal carcinogenesis and progression are largely unknown. Numerous studies have revealed that genetic and epigenetic changes and oncogenic signaling activation are the major causes of malignant transformation and progression. In recent years, the epigenetic alterations, in particular, the aberrant expression of microRNAs (miRNAs), have been shown critical roles in cancer formation, metastasis, and response to cancer therapy [1]–[6].

miRNAs are a novel class of small noncoding RNAs that typically inhibit the translation and stability of messenger RNAs (mRNAs) by binding to the 3′-untranslated regions (3′-UTR) of their target mRNAs [7]. miRNAs have 19–22 nucleotides and are found in all multi-cellular eukaryotic cells. miRNAs have important roles in various biological and pathological processes, such as development, cell proliferation, differentiation, apoptosis, inflammation, stress response and migration [1]–[3]. Increasing evidences have suggested that miRNAs are deregulated or upregulated in all types of cancers, acting either as tumor suppressors (e.g. miR-34, miR-15/16, let-7, miR 200 family) or as oncogenes (e.g. miR-155, miR-222/221, miR-17-5p, miR-21) [1], [3], [8], in which the miRNAs play key roles in important aspects of tumorigenesis, such as cancer initiation, differentiation, growth and progression [3], [5], [8], mainly by interfering with the expression of target genes involved in cell cycle, apoptosis, cell migration and invasion, angiogenesis. Using a miRNA array profile we have found that miRNA were differential expressed in colonic epithelial cells of a colorectal cancer mouse model, the Muc2 gene knockout mice [9]. One of the most changed miRNAs was miRNA-27a (miR-27a).

MiR-27a is located at chromosome 19 [10]. Its expression levels and biological functions in cancers are controversial. For instance, several studies have reported that miR-27a acts as an oncogene, whose expression is upregulated in breast cancers [11], [12], colon cancer cell lines [13]–[15], and in hepatocellular adenocarcinoma cells [16], and that the increased expression of miR-27a is associated with breast cancer progression and poor outcomes [11], [12]. Several studies have also observed that miR-27a exhibited oncogenic activity by directly suppressing ZBTB10/RINZF expression [10], [17], resulting in upregulation of transcription factor specificity protein (Sp), vascular endothelial growth factor (VEGF) and VEGF receptor 1 (VEGFR1). In another hand, miR-27a has also shown tumor suppressor roles, such as miR-27a is downregulated in esophageal cancers [18], oral squamous cell carcinoma [19], acute leukemia [20], and in non-small cell lung cancer (NSCLC) [21]. In NSCLC, miR-27a directly targets MET and EGFR 3′ UTR, leading to reduced expression of MET and EGFR [21].

This study was to determine the expression of miR-27a and association with colorectal cancer formation, progression and the underlying mechanisms. We found that miR-27a was significantly reduced in human colorectal cancer tissues and in colorectal cancer cell lines. Using the approaches of miRNA array, systemic biology, *in vitro* manipulating expression of miR-27a and *in vivo* tumor-bearing mouse model, we found that miR-27a acted as a tumor suppressor in colorectal cancer, which was through targeting SGPP1 and Smad2.

## Materials and Methods

### Ethics Statement

The animal care and use were approved by the Institutional Animal Care and Use Committee of Xinxiang Medical University and University of Illinois at Chicago, and human samples collection and use were approved by the Institutional Review Board of Xinxiang Medical University. All patients gave informed consent in written.

### Muc2 mouse colonic epithelia cells collection and miRNA array

As reported previously [9], [22], the *Muc2−/−* and *Muc2+/+* mice were generated by crossmating the *Muc2+/−* mice, and the *Muc2−/−* mice spontaneously developed colorectal tumors at age about 3 months. Thus, mouse colonic epithelial cells were collected from 3-month aged *Muc2+/+* and *Muc2−/−* mice, respectively. Four mice from each group were used. The total RNAs were extracted for miRNA array analysis. The miRNA array was performed in the Genomic Facility of University of Chicago (Chicago, Illinois). Affymetrix GeneChip miRNA Arrays version 3.0 was used for miRNA profile. The detailed experimental design, detailed protocol and data analysis could be accessed at Gene Expression Omnibus (GEO) (Access #GSE56577) and as published recently by us [23]. The animal use protocol was approved by both the University of Illinois at Chicago and Xinxiang Medical University Animal Care Committee.

### Human samples collection

Forty-one paired human colorectal cancer tissues and their adjacent normal colonic mucosa were collected from November, 2012 through October, 2013, from the First Affiliated Hospital and the Affiliated Xinxiang Central Hospital, Xinxiang Medical University. Portion of the samples were snapped into liquid nitrogen and then stored at −80°C for RNA and protein extraction for quantitative RT-PCR (qRT-PCR) and western blotting analysis, respectively. Portion of the samples were fixed in 10% buffered formalin and embedded in paraffin. Patient clinical and histopathological information were summarized in **Table 1**. The sample collection was approved by the Institutional Review Board of Xinxiang Medical University. All patients gave informed consent in written.

**Table 1:** The correlation between miR-27a levels and clinicopathological features.

Clinicopathologic Variables	N	miR-27a expression			P value
		Low (n)	High (n)		
**Gender**					
Male	23	12		11	
Female	17	7		10	>0.05
**Age**					
≤60	15	7		8	
>60	25	12		13	>0.05
**Smoking**					
Yes	10	5		5	
No	30	15		15	>0.05
**Drinking**					
Yes	7	3		4	
No	33	17		16	>0.05
**Colitis**					
Absence	20	11		9	
Presence	20	9		11	>0.05
**Tumor size**					
≤5	24	11		13	
>5	16	8		8	>0.05
**Lymphatic metastasis**					
Yes	14	8		6	
No	26	11		15	>0.05
**Lymphatic number**					
Zero	26	11		15	
Multiple (≥1)	14	8		6	>0.05
**Distant metastasis**					
Yes	12	9		3	
No	28	10		18	**0.037**
**TNM stages**					
Stage II	11	5		6	
Stage III/IV	29	14		15	>0.05
**Clinical stages**					
1–2	21	5		16	
3–4	19	14		5	**0.00016**
**Differentiation**					
Low/Moderate	12	7		5	
High	28	13		15	>0.05

N, number of patients in this study; n, number of patients in each group.

### Immunohistochemical staining

Formalin-fixed and paraffin-embedded tissues were sectioned into 4-micron thickness. The detailed procedures of immunohistochemical staining were similar as we reported in our publications [24]–[26]. The first antibodies used for staining were anti-SGPP1 (Abcam, Cambridge, MA), anti-Smad2 and anti-phosphorylated STAT3 (Cell Signaling Technologies, Danvers, MA).

### Cell culture

Human colorectal cancer cell lines HCT116, Caco 2 and SW480 from ATCC were maintained in a complete MEM medium. Human normal colon mucosal epithelial cell line NCM460 was purchased from the Shanghai Bioleaf (Shanghai, China) and maintained in RPMI-1640 medium. The murine colon adenocarcinoma cell line MC38 (derived from C57BL6/J mouse) [27] was cultured in MEM (Invitrogen, Carlsbad, CA). All media were supplemented with 10% FBS and antibiotics (10,000 U/ml penicillin, 10 µg/ml streptomycin). Cells were cultured at 37°C in a humidified atmosphere containing 5% CO_2_.

### MiR-27a transfection

miR-27a precursor and miR-27a mimics were purchased from Shanghai GenePharma (Shanghai, China). HCT116, Caco 2 and SW480 cells were seeded in six-well plates at 2×10^5^, 1.2×10^5^ and 4×10^5^ per well, respectively. Twenty-four hours after plating, 4.0 µg of miR-27a precursor were transfected to the cells with Lipofectamine (Invitrogen, Carlsbad, CA) following the manufacture’s protocol. Negative miR-27a precursor (GenePharma, Shanghai) was also transfected as negative controls.

### MiR-27a targets prediction

The online software TargetScan (www.targetscan.com), starBase (http://starbase.sysu.edu.cn/), Tarbase (http://microrna.gr/tarbase/), and miRbase (http://mirbase.org/index.shtml) were used to predict miR-27a targets. The targets were clustered by biological functions using blast2go (GO) tool (http://www.blast2go.com/b2ghome).

### RNA extraction and quantitative RT-PCR for miRNA and mRNA analysis

Trizol reagent (Invitrogen, Carlsbad, CA) was used for total RNA extraction from the frozen colorectal cancer and normal tissues following manufacturer’s protocol. Quantitative RT-PCR (qRT-PCR) (Applied Biosystem Inc.) was used for miRNA and mRNA quantification analysis. The primers for miR-27a analysis were: reverse transcription (RT) primer: GTGCAGGGTCCGAGGTCAGAGCCACCTGGGCAATTTTTTTTTTTGCGGAA; forward primer: TTCGGTTCACAGTGGCT AAG; internal control snord47 primers were: RT primer: GTGCAGGGTCCGAGGTCAGAGCCACCTGGGCAAT TTTTTTTTTTaacctc; forward primer: CGCCAATGATGTAATGATTCTG; Universal reverse primer: CAGTGCAGGGTCCGAGGT. Universal Taqman probe: 56-FAM/CAGAGCCAC/ZEN/CTGGGCAATTT/3IABkFQ. The primers for SGPP1 mRNA analysis were: forward primer: TGGTCCTC CTCACCTATGGC; reverse primer: CTAGAGAACACCAGCAGGGA. The primers for Smad2 mRNA analysis were: forward primer: AACAGAACTTCCGCCTCTGG; reverse primer: GGAGGTGGCGTTT CTGGAAT. The primers for internal control GAPDH mRNA were: forward primer: GTCAAGGCTGAGAACGGGAA; reverse primer: AAATGAGCCCCAGCCTTCTC.

### Dual luciferase report construction and transfection

The full length of 3′-UTR of SGPP1 and Smad2 were cloned from human genomic DNA and inserted into psiCHECK-2 vector (Promega, Madison, WI), to generate psiCHECK-2-3′-UTR-SGPP1 and psiCHECK-2-3′-UTR-Smad2 luciferase reporter system, respectively. Twenty-four hours before transfection, 1.2×10^4^ cells were seeded in a 96-well plate. In brief, 10 pmol of miR-27a mimics or negative miRNA mimics (negative control) was co-transfected into cells with 100 ng of psiCHECK-2-3′-UTR-SGPP1 or psiCHECK-2-3′-UTR-Smad2, respectively, using DharmaFect Duo reagent (Dharmacon, Lafayette, CO, USA). Luciferase assay was performed 24 h after transfection by the dual-luciferase reporter assay system (Promega, Madison, WI). For each sample, firefly luciferase activity was normalized to Renilla luciferase activity and the inhibition of miR-27a on SGPP1 3′-UTR and Smad2 3′-UTR was normalized to the control mimics.

### Cell proliferation assay

The MTS assay was used as a cytotoxicity assay for the HCT116 cells transfection with miR-27a using the Lipofectamine 2000 (Invtrogen, Carlsbad, CA). Briefly, 1×10^5^ cells were seeded into 96-well plate transfected with 0.2 µg of miR-27a precursor with 0.5 µl Lipofectamine (Invitrogen, Carlsbad, CA). After 24, 48 and 72 hours, cell proliferation was determined by MTS assay (3-(4,5-dimethylthiazol-2-yl) 5-(3-carboxymethoxyphenyl)-2-(4-sulfophenyl)-2H-tetrazolium) according to the manufacturer’s protocol (CellTiter 96 Non-Radioactive Cell Proliferation Assay Kit, Promega Corporation, Madison, WI). The remaining viable cells with MTS uptake were determined by measuring the optical density at 570 nm using an enzyme-linked immunosorbent assay reader (Molecular Devices, Sunnyvale, CA). Values shown were mean +/− standard deviation. At least three measurements were read, and the experiments were conducted 3 times independently.

### Apoptosis analysis

To detect apoptosis, the HCT116 cells were transfected with miR-27a precursor or negative control miRNA precursor. After 48 hours, the cells were harvested and fixed with 70% ethanol followed by propidium iodide (P.I.) and Annexin V (Invitrogen, Carlsbad, CA) staining. Cells were then counted by flow cytometry (FACScan, BD Biosciences, San Jose, CA) for apoptosis analysis. Usually, about 10,000 cells were counted. The percentage of the apoptosis was calculated by dividing the total cells by apoptotic cells.

### Wound healing assay

As reported previously [26], the HCT116 cells transiently transfected with miR-27a precursor or negative control miRNA precursor were seeded in a 100-mm Petri dish. A wound was made by scratching on the Petri dish bottom, followed by another 48 hours growth.

### Immunoblotting

For immunoblotting, the human colon cancer cells HCT116 and SW480 were collected 72 hours after transfection with miR-27a precursor or negative control miRNA precursor. Cells were lysed using 1x RIPA buffer (Upstate Biotechnology, Lake Placid, NY) containing a protease inhibitor cocktail (Sigma, St. Louis, MO). After cell lysis, 45 µg of protein was loaded on a 10% SDS gel followed by transfer to PVDF membrane. Antibodies against SGPP1 (Abcam, Cambridge, MA), Smad2, Stat3, phosphorylated Stat3 and Caspase 3 (Cell Signaling Technologies, Danvers, MA), and β-actin (Sigma, St. Louis, MO) were used. Secondary antibody was purchased from Santa Cruz Biotechnology (Santa Cruz, CA). The detected signals were visualized by an enhanced chemiluminescence kit (Beyotime Institute of Biotechnology, Haimen, Jiangsu, China), as recommended by the manufacturer.

### Tumor-bearing (Xenografts) study

As reported recently [26], 1.5×10^5^ murine colon adenocarcinoma cells MC38 re-suspended in 150 µl PBS were injected subcutaneously into the flank of the normal C57B/l6 mice at age about 8 weeks (5 mice per group). Both MC38 cell line and the mice were in C57BL/6 background and no rejection occurred. The animals were maintained in a pathogen-free barrier facility and closely monitored by animal facility staff. The grown tumors (xenografts) were measured every 3 day starting 21 days post inoculation of MC38 cells using caliper as length x width x width/2 (mm^3^). 6.26 µg of miR-27a precursor or negative miRNA (GenePharma, Shanghai, China) mixed with 1.6 µl transfection reagent Lipofectamine 2000 (Invitrogen) in 50 µl PBS were injected into the tumors every 3 days, for total of 3 times. 30 days after inoculation, the animals were sacrificed and the xenografts were isolated, the weight (gram) and volume (mm^3^) of the xenografts were determined. All procedures were conducted according to the Animal Care and Use guideline approved by Xinxiang Medical University Animal Care Committee.

## Results

### MiR-27a expression was reduced in *Muc2−/−* mouse colonic epithelial cells, in human colorectal cancer tissues and colorectal cancer cells

**Figure 1:**
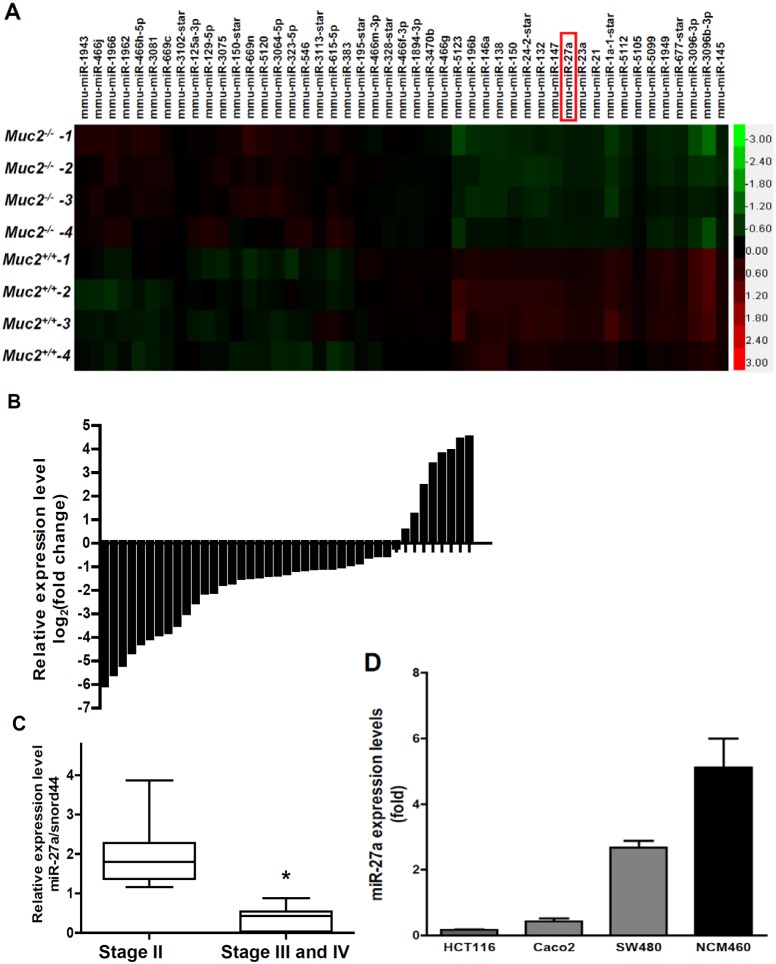
miR-27a was down-regulated in Muc2−/− colonic epithelial cells and human colorectal cancers. A, Cluster analysis of miRNAs expression profiles in mouse colonic epithelial cells from *Muc2−/−* mice and *Muc2+/+* mice (4 mice each group). Upregulated expression was indicated in red, whereas downregulated expression was coded in green. B, miR-27a was frequently downregulated in human colorectal cancers (30 of 41 cancers exhibited downregulation and 11 cases showed upregulation, in comparison to the adjacent normal tissues). Expression levels of miR-27a were normalized to the corresponding levels of SNORD44. Data were analyzed using a ΔΔCt approach and expressed as log2 fold change (ΔΔCt [cancer/normal]). C, Differential expression of miR-27a at stage II and stage III/IV cancers. The difference was significant using Mann Whitney test (*p<0.0001). D, miR-27a expression was reduced in colorectal cancer cell lines, compared to the immortalized normal colon epithelial cell (NCM460).

Our previous studies have demonstrated that *Muc2−/−* mice spontaneously developed colorectal cancers and the carcinogenesis is linked to chronic inflammation [9], [22], [28], [29]. Interestingly, chronic colitis is frequently seen at the early age of *Muc2−/−* mice, and colorectal tumors are frequently seen after 3 months of age. Thus, the 3 months could be a borderline of the transition from chronic inflammation to tumor formation. To indentify the potent miRNAs involving in the malignant transformation, we isolated colonic epithelial cells from the age of 3 months mice, extracted RNA and performed miRNA profile. As shown in Figure 1A, miRNA profile showed differential expression of miRNAs in both *Muc2−/−* and *Muc2+/+* mouse colonic epithelial cells. Based on potential biology roles in proliferation and inflammation, miRNA profile and published literatures, miR-27a was chosen for further studies. Since genetic deficiency of the Muc2 gene in mice causes colorectal cancer formation, the decreased expression of miR-27a in colonic epithelial cells could be involved in the carcinogenesis.

To determine the role of miR-27a in colonic epithelial cell malignant transformation, miR-27a expression levels were determined in human colorectal cancer and their adjacent tissues. In comparison with the adjacent normal colon mucosa, 73% (30/41) of colorectal cancer tissues shown reduced miR-27a expression, and only 27% (11/41) colorectal cancers exerted upregulated expression of miR-27a, the difference was significant (Figure 1B, p<0.01). More importantly, the reduced expression of miR-27a was also associated with colorectal cancer pathological stages – miR-27a levels were more downregulated at stages III/IV than those at stage II (Figure 1C, p<0.0001). In addition, reduced miR-27a was correlated with distant metastasis (Table 1).

The expression levels of miR-27a were further determined in human colorectal cancer cell lines. As shown in Figure 1D, miR-27a expression was downregulated 95%, 90% and 52% in HCT116, Caco-2 and SW480 cells, respectively, compared to the immortalized normal human colon epithelial cell NCM460.

### miR-27a targets prediction

Above findings strongly suggested miR-27a was frequently downregulated in colorectal cancers. Therefore, it is essential to determine whether the reduction of miR-27a is involved in colorectal cancer formation and progression, and it is warranted to identify miR-27a targets and reveal the underlying molecular mechanisms. Previous studies have demonstrated that ZBTB10/RINZF is a direct target of oncogenic miR-27a in breast cancers [10], [17] and colon cancer cell lines [15], but this could not be the case in the colorectal cancer tissues because herein miR-27a seemed to be a tumor suppressor in colorectal cancers. We employed multiple tools to predict novel targets of miR-27a. Supplemental Figure S1A (Supplemental materials) showed 346 potent targets at different signaling pathways, among them, the categories of cell cycle, cell death, cell differentiation, cell growth and proliferation and cellular homeostasis, etc, could be more relevant to cancer development and progression. We further narrowed down the targets using GO program, and the targets at the categories of cellular process, single organism process, biological regulation and metabolic process, etc, exhibited stronger scores, meaning more relevant to miR-27a-associated functions in cancer formation and progression (Supplemental Materials Figure S1B).

### SGPP1 and Smad2 were the targets of miR-27a

Among the hundreds of targets, the two novel targets of miR-27a, Sphingosine-1-phosphate phosphatase 1 (SGPP1) and Smad2, were chosen for further studies. SGPP1 is a catalyze of Sphingosine-1-phosphate (S1P), the later is a bioactive sphingolipid metabolite that regulates diverse biologic processes [30], [31], and recent report showed that SIP is linked to signal transducer and activator of transcription 3 (STAT3) activation and the development of colitis-associated colorectal cancer [31]. But the biological role of SGPP1 on carcinogenesis is not clear. Smad 2 is a member of Smad family and is associated with cell growth and proliferation through transforming growth factor β (TGF-β) signaling pathway [32], [33].

**Figure 2:**
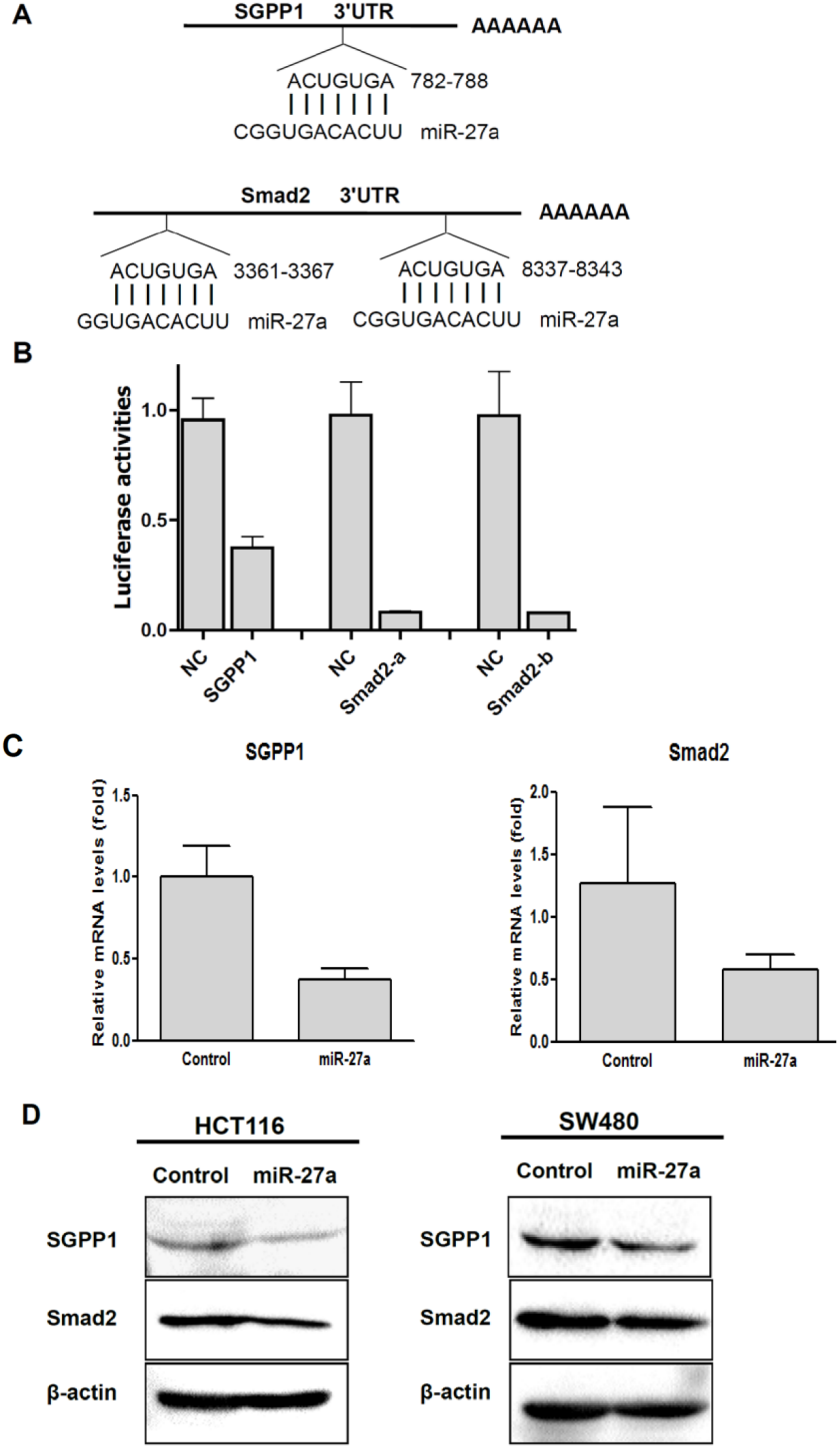
miR-27a targeted SGPP1 and Smad2. A, There was one miR-27a binding site at SGPP1 3′-UTR, and there were two miR-27a binding sites at Smad2 3′-UTR. B, miRNA-27a suppressed SGPP1 and Smad2 reporter activities assayed by Dual Luciferases in HCT116 cells. (NC, negative control). C, miRNA-27a inhibited SGPP1 and Smad2 mRNA expression in HCT116 cells. D, miR-27a suppressed SGPP1 and Smad2 protein in colon cancer cells HCT116 and SW480.

Genomic alignment showed that 3′-UTR of SGPP1 and Smad2 have one or two miR-27a binding sites (Figure 2A). To determine whether miR-27a could repress SGPP1 and Smad2 expression by targeting its binding site at 3′-UTR in SGPP1 and Smad2, the PCR products containing full length of 3′-UTR with intact target site of miR-27a recognition sequences were inserted into the luciferase reporter vector. The plasmids were transfected into HCT116 cells that had been transfected with control miRNA mimics or miR-27a mimics, and the luciferase reporter activities were measured. As shown in Figure 2B, SGPP1 reporter activities were reduced about 65% and both of the Smad2 reporter activities (Smad2-a and Smad2-b) were reduced about 90%, respectively. These finding confirmed that SGPP1 and Smad2 were the targets of miR-27a.

The repression of SGPP1 and Smad2 expression by miR-27a were determined by qRT-PCR and immunoblotting. As shown in Figure 2C, SGPP1 and Smad2 mRNA levels were repressed about 60% and 50% by miR-27a in HCT116 cells, respectively, consistent with the repression on their reporter luciferase activities. SGPP1 and Smad2 protein levels were also downregulated by miR-27a on both HCT116 and SW480 colorectal cancer cells (Figure 2D).

### SGPP1 and Smad2 were inversely correlated with miR-27a in colorectal cancer

**Figure 3:**
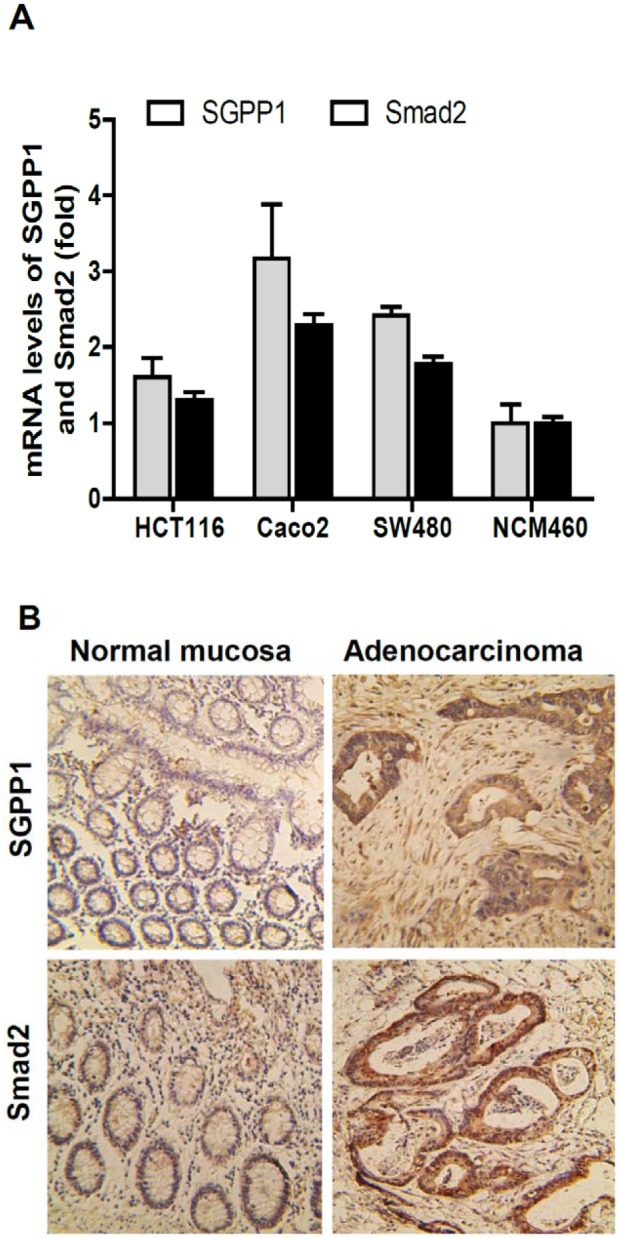
SGPP1 and Smad2 were overpressed in human colorectal cancer cells and cancer tissues, which were inversely correlated with miR-27 expression. A, SGPP1 and Smad2 mRNA levels were increased in colon cancer cell lines, compared to the normal colon epithelial cell (NCM460), assayed by qRT-PCR. B, SGPP1 and Smad2 were overexpressed in colorectal cancers, compared to adjacent normal mucosa, assay by immunohistochemical staining.

As SGPP1 and Smad2 were likely the targets of miR-27a, we then determined the expression levels of SGPP1 and Smad2 in human colorectal cancers and cancer cell lines. As shown in Figure 3A, SGPP1 mRNA levels were 1.6-, 3.2- and 2.4-fold in HCT116, Caco-2 and SW480 cells, respectively, than that in the normal colonic epithelial cell NCM460 cells. Similar as the expression levels of SGPP1, Smad2 mRNA levels were also upregulated in HCT116, Caco-2 and SW480 cells, showing 1.4-, 2.3- and 1.8-fold higher than that in the normal colonic epithelia NCM460 cells (Figure 3A). Both SGPP1 and Smad2 mRNA levels were inversely correlated with the miR-27a levels at these human colorectal cancer cells (Figure 1D).

The expression of SGPP1 and Smad2 were then evaluated in human colorectal cancers by immunohistochemical staining. Compared to the adjacent normal colon mucosa, both SGPP1 and Smad2 expression were much higher at colorectal adenocarcinomas (Figure 3B), negatively correlated with miR-27a levels in colorectal cancers in which miR-27a was frequently reduced (Figure 1B).

### MiR-27a inhibited cell proliferation, enhanced apoptosis and attenuated cancer cell migration

**Figure 4:**
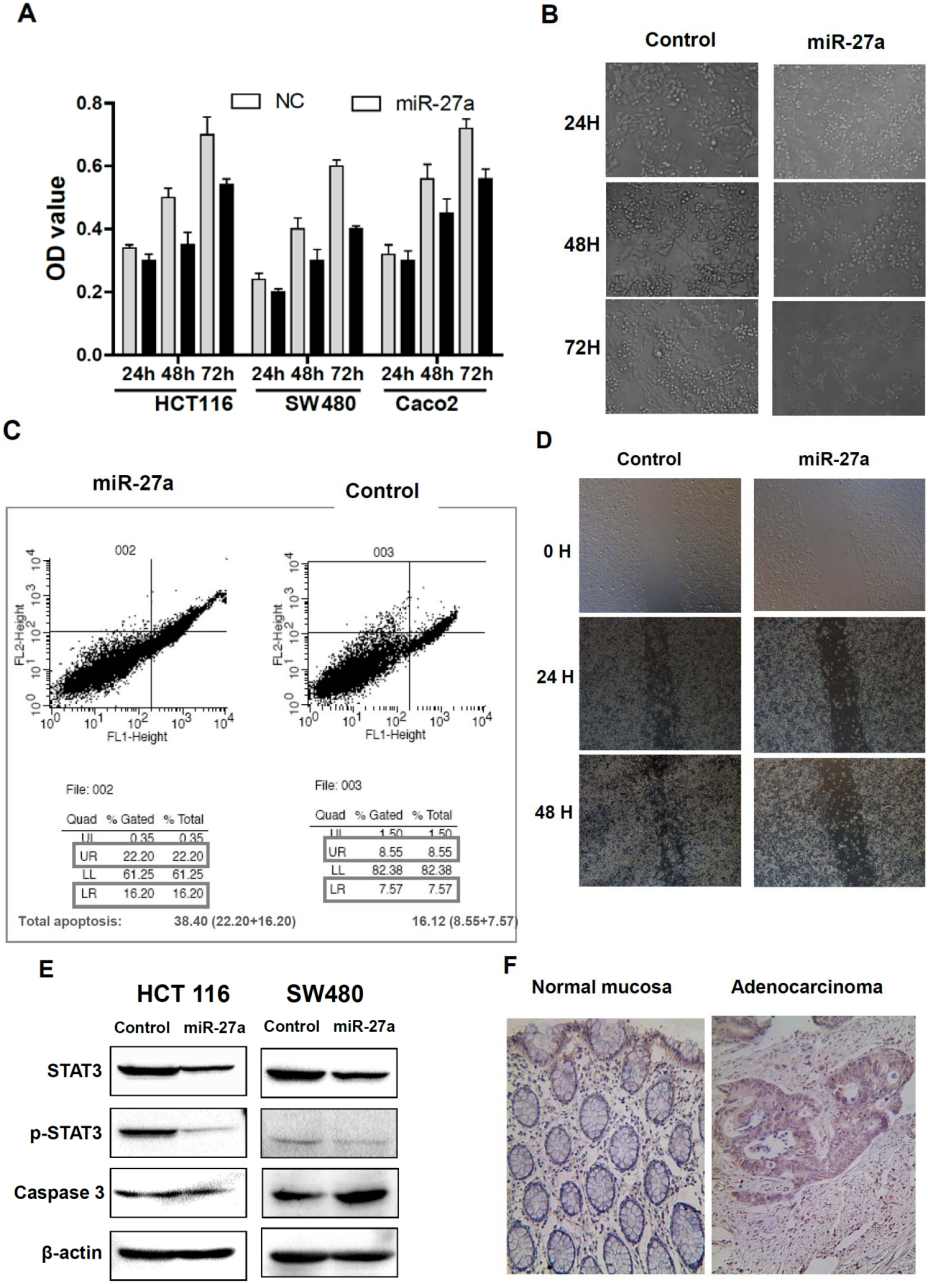
miR-27 tumor suppressor functional studies. A, miR-27a inhibited cancer cell proliferation at SW480, HCT116 and Caco 2 cells, assayed by MTS. (NC, negative control). B, Examples of cell proliferation inhibition at HCT116 cells. C, miR-27a enhanced apoptosis, assayed by flow cytometry. The percentage at UR (upper right area) and LR (lower right area) stood for the total apoptosis (UR + LR). D, miR-27a attenuated cancer cell migration in HCT116 cells, assayed by wound healing. E, miR-27a affected STAT3 and Caspase3 levels in HCT116 and SW480 cells. F, p-STAT3 were overexpressed in colorectal cancers, compared to adjacent normal mucosa, assay by immunohistochemical staining.

To determine miR-27a tumor suppressing functions, miR-27a precursor were transfected into the HCT116 cell, and the effects on cell proliferation, apoptosis and migration were analyzed. As shown in Figure 4A and 4B, increasing miR-27a significantly inhibited cancer cell proliferations after 48 and 72 h at HCT116, Caco-2 and SW480 cells, although the effects at 24 h post transfection were not changed. In addition, increasing miR-27a significantly enhanced cancer cell apoptosis (45% at miR-27a groups versus 15% at vector control group, p<0.01). The examples of apoptosis changes were shown at Figure 4C.

Moreover, increased miR-27a significantly attenuated HCT116 cancer cell migration at 24 and 48 h after transfection, assayed by wound healing (Figure 4D).

To determine the mechanisms of miR-27a mediated inhibition of cell proliferation and migration and enhancement of apoptosis, we determined the changes of STAT3 and Caspase3. We found that besides the downregulation of total STAT3 by miR-27a, the phosphorylated STAT-3 (p-STAT3) was also dramatically repressed by miR-27a (Figure 4E). Since miR-27a was reduced in human colorectal cancers, we determined whether p-STAT3 was increased in colorectal cancers. P-STAT was indeed increased in colorectal cancers (Figure 4F).

### MiR-27a inhibited cancer growth in mice

**Figure 5:**
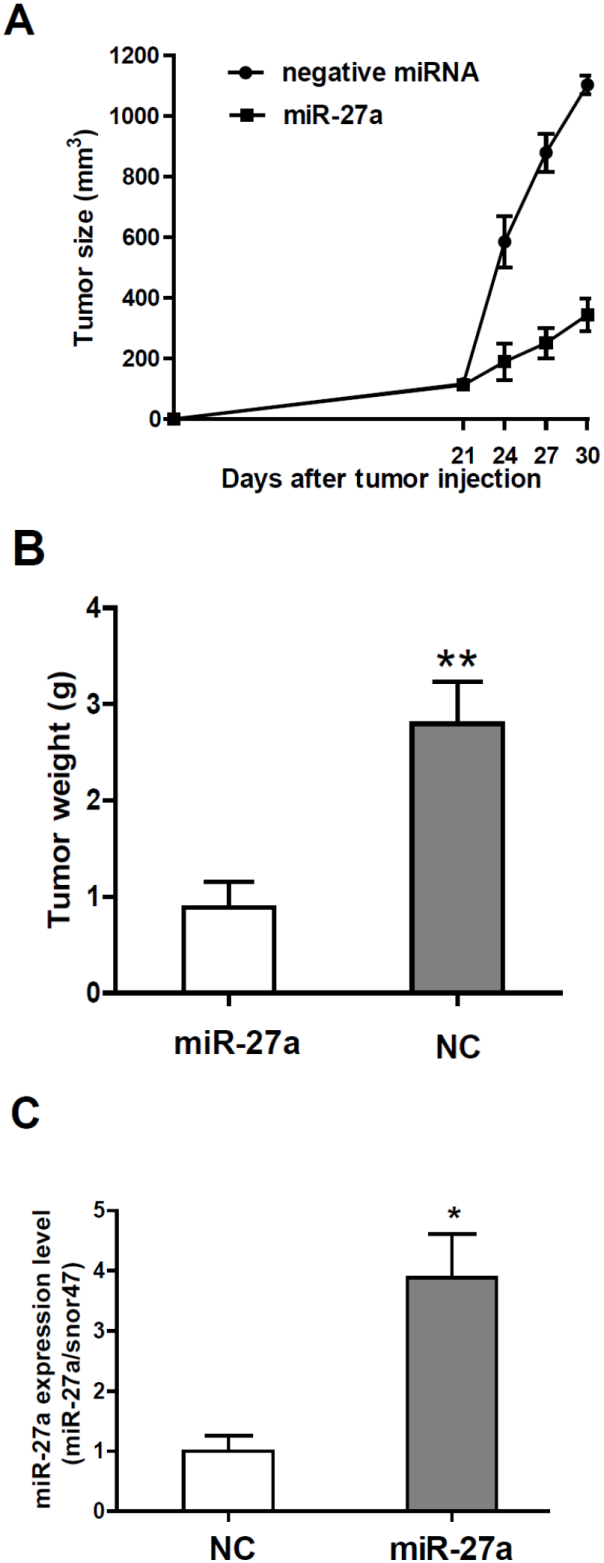
miR-27a inhibited murine colon cancer cell MC38 growth *in vivo*. A, miR-27a suppressed tumor growth. When xenografts were palmbable at day 21, a mixture of miR-27a precursor and Lipofectamine 2000 was directly injected into the tumors. Tumor sizes were measured every three days. B, Tumor weights were significantly reduced in miR-27 treated groups compared to the untreated group (NC). (**p<0.01, compared to the untreated group.) C, miR-27 levels in tumors from untreated (NC) and miR-27a treated groups were validated by qRT-PCR. (*p<0.05, compared to the untreated group).

To determine miR-27a tumor suppressing effects *in vivo*, we transplanted murine colon cancer cells MC38 into wild-type C57Bl/6 mice and injected a mixture of miR-27a precursor and Lipofectamine 2000 into the tumors when the tumors were palmable at day 21 post inoculation. Compared to vector control, miR-27a treatment significantly inhibited cancer cell growth in mice, in terms of significant reduction of tumor sizes and weight (Figure 5A, 5B). qRT-PCR showed that miR-27a level was still at a higher level in the tumors isolated from mouse xenografts (Figure 5C).

## Discussion

It is well known that the functions and target genes of a miRNA are tissue- and cancer-type specific. Functional studies have shown that miR-27a has shown both oncogenic and tumor suppressive functions in different cell lines and human cancer tissues. For example, miR-27a directly suppresses ZBTB10/RINZF expression [10], [17]. and in turn upregulates VEGF and VEGF receptor in the cancers of breast [11], [12] and colon [13]–[15], and overexpression of miR-27a is associated with poor outcomes [11], [12]. Whereas, in the cancers of esophagus [18], oral cavity [19], lung [21], and head and neck [34], miR-27a is downregulated, and miR-27a directly targets MET and EGFR and suppresses their expression in lung cancer [21]. In this study, we found that miR-27a was significantly downregulated in colorectal cancers and colorectal cancer cells. Importantly, the downregulated miR-27a was also associated with colorectal cancer pathological stages and distant metastasis, showing tumor suppressor roles in colorectal cancer. First, *in vitro* studies showed that increased expression of miR-27a inhibited colorectal cancer cell proliferation, promoted apoptosis and attenuated cancer cell migration. Second, in a tumor-bearing mouse model, a direct injection of miR-27a to tumor suppressed tumor growth. These findings strongly suggested that miR-27a could be used as a biomarker to monitor cancer development and progression, and could be used as a potential therapeutic target and even a potential therapeutic agent for colorectal cancer.

The mechanistic studies further showed that miR-27a-mediated tumor suppressor could be through targeting SGPP1, Smad2 and STAT3. Previous studies have demonstrated that Apc mutation and Wnt/β-catenin signaling activation, and chronic inflammation in colon, are the two major causes for colorectal cancer formation [35]–[37]. The studies from us have shown that genetic deletion of the Muc2 gene is sufficient to cause chronic colitis and rectal inflammation at early stage, and leads to colorectal cancer formation at late stage [9], [22], [29], [38]. The malignant transformation is linked to the activation of inflammatory signaling and upregulation of cytokines [22], [38]. And the chronic inflammation facilitated Apc-mutation-caused gastrointestinal tumor formation in the Apc/Muc2 double gene knockout mouse model of colorectal cancer [22]. In fact, other studies have demonstrated that the transcription factor STAT3 also plays a critical role in inflammation-associated colorectal cancer formation [31], [39]–[42]. During the transition of chronic intestinal inflammation to colitis-associated cancer, STAT3 could be persistently activated by sphingosine-1-phosphate (S1P) produced by upregulation of sphingosine kinase 1 (SphK1), which was linked to production of the multifunctional NF-kB-regulated cytokine IL-6, and consequently upregulating of the S1P receptor, S1PR1 [31]. While, the balance of S1P levels are regulated by sphingosine-1-phosphate phosphatase 1 (SGPP1) [30].

Using comprehensive approaches, including qRT-PCR, immublotting and in situ immunohistochemical staining, we showed that the expression of SGPP1 at mRNA and protein levels were upregulated in colorectal cancers and colorectal cancer cell lines, which were inversely correlated with the expression of miR-27a. Both dual luciferase assay and increasing expression of miR-27a further showed the inhibitory effects of miR-27a on SGPP1. Thus, this is the first to reveal that SGPP1 is a potent direct target of miR-27a, although the evidence of direction regulatory interaction is needed for further investigation. Limited studies have demonstrated that SGPP1 catalyzes the degradation of S1P via salvage and recycling of sphingosine into long-chain ceramides, and that aberrant expression of SGPP1 is associated with idiopathic pulmonary fibrosis, and pulmonary fibrosis [30]. However, the biological functions for SGPP1 in carcinogenesis are largely unknown and worthy further elucidative.

Smad2 is a member of the Smad family and is a key element in TGF-β signaling. Therefore, accompanying actions of TGF-β in different circumstances, e.g., in regulating development and differentiation in physiological cell process, and in facilitating cell growth and migration and angiogenesis in cancers, Smad2 exerts dual functions as tumor suppressor or oncogene [32], [33], [43], [44]. In the current study, we found that, similar as SGPP1, Samd2 expression at mRNA and protein levels was upregulated in colorectal cancers and cell lines, exhibiting oncogenic phenotypes; moreover, Smad2 was repressed at transcriptional and translational levels by miR-27a, suggesting the direct target of miR-27a, a novel finding that has not been reported previously. A few recent studies have reported that Smad2, similar as Smad3 and Smad4, is mutant in cancers [43], but Smad2 plays differential roles from Smad3 and Samd4 in TGF-β signaling [33]. The mutation frequency in colorectal cancer and whether the mutation of Smad2 leads to stability of SMAD2 protein in the colorectal cancer is not clear and warrants further investigation. Previous studies have also shown therapeutic role of Smad2, that blocking Smad2 could suppress TGF-β-induced tumorigenesis, epithelial-mesenchymal transition (EMT), cell motility, and invasion [45], indicating that targeting miR-27a/Smad2 could have a great impact on developing a novel strategy for colorectal cancer therapy. The new signal pathway of miR-27a-Smad2-TGF-β could also contribute to the inhibitory role of miR-27a on cancer cell migration (Figure 4D), invasion and metastasis, detailed mechanism is under investigation.

In summary, we have demonstrated that miR-27a is frequently downregulated in colorectal cancer, and the reduced miR-27a is correlated with cancer distant metastasis and histopathological stages, and thus, miR-27a acts as a tumor suppressor. Both *in vivo* and *in vitro* studies have identified SGPP1 and Smad2 as two novel targets of miR-27a, which is linked to STAT3 to regulate cancer cell proliferation, apoptosis and migration. Therefore, miR-27a could be a useful biomarker for colorectal cancer development and progression, and also could have a therapeutic potential targeting SGPP1, Smad2 and Stat3 for colorectal cancer therapy.

## Supporting Information

Figure S1 **Prediction of miR-27a targets.** A. The targets were categorized by biological process (filtered by sequences numbers, cutoff = 5.0). B, The targets were clustered into multiple categories by biological process level 2 using Go Oncology tool.
(TIF)
