# Expression of Concern: Tumor Suppressor MicroRNA-27a in Colorectal Carcinogenesis and Progression by Targeting SGPP1 and Smad2

**DOI:** 10.1371/journal.pone.0280980

**Published:** 2023-01-26

**Authors:** The PLOS ONE Editors

Following the publication of this article [1], concerns were raised regarding the reported methodology and results. Specifically,

- The Fig 2D HCT116 and SW480 β-actin panels appear similar to the Fig 4E HCT 116 and SW480 β-actin panels respectively.
- The Fig 4D Control 24H results appear to partially overlap with the Fig 4D Control 48H results.
- The article does not report the miR mimic and control sequences used in the study.

In addition, concerns were raised regarding similarities between the Figs 1, 2, 3, 4 and 5 presented in this article [1] and the Figs 1, 3, 4, 5 and 6 presented in an unrelated study [2, retracted in 3] published by a different research group. This second study [2, retracted in 3] was submitted to *Cancer Gene Therapy* after the *PLOS ONE* article [1] was published. The corresponding author states that they were unaware of the publication of [2, retracted in 3].

The corresponding author stated that the cell migration assays reported in Fig 4D partially overlap because the images were obtained from the same petri dish at different time points. In addition, the corresponding author clarified that the Fig 2D and Fig 4E results were obtained simultaneously from the same lysate, using the same blot probed with multiple antibodies. Furthermore, the corresponding author clarified that the Fig 4B results represent examples of cell proliferation inhibition at SW480 cells, as opposed to the reported HCT116 cells. The legend for Fig 4B should read: B, Examples of cell proliferation inhibition at SW480 cells.

The corresponding author provided the following information regarding the miRNA mimic and control sequences that are missing from the study: The sequence for the miR-27a mimics (GenePharma Company, Shanghai) is UUCACAGUGGCUAAGUUCCGC; the sequence for the negative miRNA mimics (negative control) (GenePharma Company, Shanghai) is UUGUACUACACAAAAGUACUG.

In addition to the issues listed above, editorial review of the other primer sequences reported in this study and the primer sequence information provided by the corresponding author indicated that the sequence for the SGPP1 mRNA analysis reverse primer was reported incorrectly, and this sequence should read ACTAGAGAACACCAGCAGGGA instead. Furthermore, regarding the primers provided for the miR-27a and snord47 primers, the corresponding author clarified that the primer design for the miRNA analysis was based on the S-Poly(T) miRNA assay approach, which utilizes an S-Poly(T) primer that includes an oligo(dT)11 sequence, followed by 5–7 miRNA-specific bases, permitting higher RT efficiency, and thus better sensitivity and specificity than the poly(A) and stem-loop methods [4].

The data used to prepare the Fig 1A miRNA microarray results are publicly available on the NCBI Gene Expression Omnibus database, query DataSet GSE56577 [5]. The authors have provided the individual level data underlying Figs 1B-D, 2BC, 3A, and 5A-C, which are available in the S1–S5 Files below. Although the article’s data availability statement reads “The authors confirm that all data underlying the findings are fully available without restriction”, the corresponding author stated that the uncropped underlying blot data used to prepare the results presented in Figs 2D and 4E are no longer available. In addition, the corresponding author clarified that the FACS experiments were conducted by a collaborator and that the authors do not have access to the raw FACS data used to prepare the Fig 4C results.

The *PLOS ONE* Editors issue this Expression of Concern to notify readers of the above issues and relay the available supporting data and additional primer information provided by the corresponding author.

## Supporting information

S1 File Available underlying data for Fig 1. (ZIP)

S2 File Available underlying data for Fig 2. (ZIP)

S3 File Available underlying data for Fig 3. (ZIP)

S4 File Available underlying data for Fig 4. (PDF)

S5 File Available underlying data for Fig 5. (ZIP)

## References

[1] Bao Y, Chen Z, Guo Y, Feng Y, Li Z, Han W, et al. (2014) Tumor Suppressor MicroRNA-27a in Colorectal Carcinogenesis and Progression by Targeting SGPP1 and Smad2. PLoS ONE 9(8): e105991. doi: 10.1371/journal.pone.0105991 25166914 PMC4148394

[2] Ruoming W, Zhen Y, Tengteng Z, et al. (2015) RETRACTED ARTICLE: Tumor suppressor microRNA-31 inhibits gastric carcinogenesis by targeting Smad4 and SGPP2. Cancer Gene Ther 22, 564–572. 10.1038/cgt.2015.41 26494556

[3] Ruoming W, Zhen Y, Tengteng Z, et al. (2021) Retraction Note: Tumor suppressor microRNA-31 inhibits gastric carcinogenesis by targeting Smad4 and SGPP2. Cancer Gene Ther 28, 545. doi: 10.1038/s41417-021-00325-5 33820947

[4] Kang K, Zhang X, Liu H, Wang Z, Zhong J, Huang Z, et al. (2012) A Novel Real-Time PCR Assay of microRNAs Using S-Poly(T), a Specific Oligo(dT) Reverse Transcription Primer with Excellent Sensitivity and Specificity. PLoS ONE 7(11): e48536. doi: 10.1371/journal.pone.0048536 23152780 PMC3496722

[5] https://www.ncbi.nlm.nih.gov/geo/query/acc.cgi?acc=GSE56577

