# Supplementary figures and images for: Tumor Suppressor MicroRNA-27a in Colorectal Carcinogenesis and Progression by Targeting SGPP1 and Smad2
Source: PLoS One. 2014 Aug 28;9(8):e105991. doi: 10.1371/journal.pone.0105991 (PMC4148394; doi:10.1371/journal.pone.0105991)

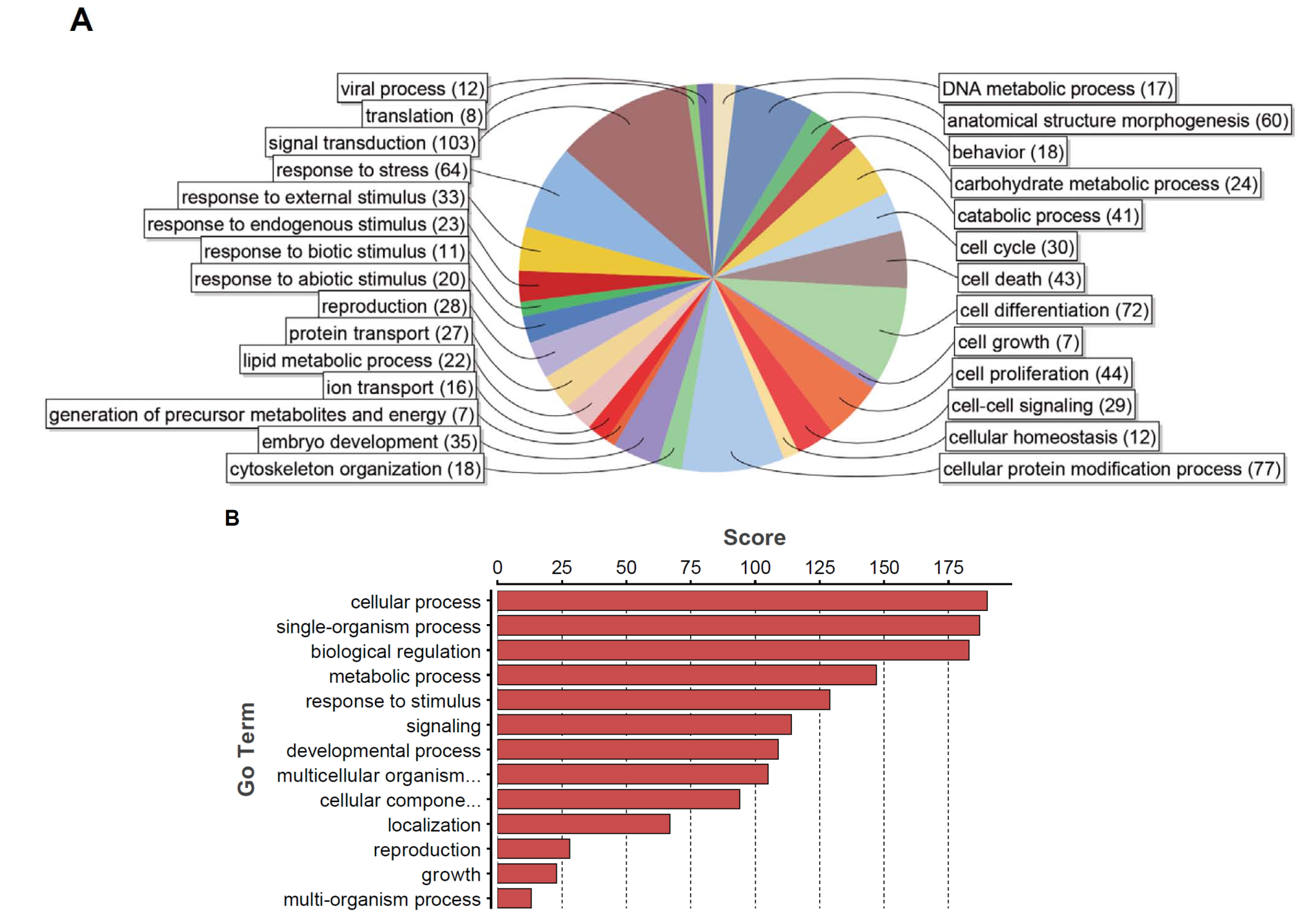

Supplement: Figure S1 — Prediction of miR-27a targets. A. The targets were categorized by biological process (filtered by sequences numbers, cutoff = 5.0). B, The targets were clustered into multiple categories by biological process level 2 using Go Oncology tool. (TIF) [file pone.0105991.s001.tif]
