# Supplementary material for: Expression of Concern: Tumor Suppressor MicroRNA-27a in Colorectal Carcinogenesis and Progression by Targeting SGPP1 and Smad2
Source: PLoS One. 2023 Jan 26;18(1):e0280980. doi: 10.1371/journal.pone.0280980 (PMC9879486; doi:10.1371/journal.pone.0280980)
Supplement: S1 File — (ZIP) [file pone.0280980.s001.zip › S1 File - Available underlying data Figure 1/Fig.1A Raw data information.docx]

**Fig.1A and supplemental Fig. S1**

**Raw data could be found in the NCBI Gene Expression Omnibus (GEO) (Access # GSE56577)**

<https://www.ncbi.nlm.nih.gov/geo/query/acc.cgi?acc=GSE56577>

**miR-27 targets prediction and their target function stratification were published as a Supplemental Material (Supplemental Materials Figure S1) (Attached file: miR-27aSupplementalFigures03082014)**

Top of Form

| \| \| GEO accession:  \| 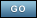 \| \| --- \| --- \| \| \| --- \| --- \| --- \| |
| --- | --- | --- | --- |

Bottom of Form

| \| \| [**Series GSE56577**](https://www.ncbi.nlm.nih.gov/geo/query/acc.cgi?acc=GSE56577) \|  \| [Query DataSets for GSE56577](https://www.ncbi.nlm.nih.gov/gds/?term=GSE56577%5bAccession%5d) \| \| --- \| --- \| --- \| \| \| \| --- \| --- \| --- \| --- \| --- \| \| Status \| Public on May 31, 2014 \| \| Title \| Differential expression of microRNA in Muc2-/- mouse colonic epithelial cells \| \| Platform organism \| [synthetic construct](https://www.ncbi.nlm.nih.gov/Taxonomy/Browser/wwwtax.cgi?mode=Info&id=32630) \| \| Sample organism \| [Mus musculus](https://www.ncbi.nlm.nih.gov/Taxonomy/Browser/wwwtax.cgi?mode=Info&id=10090) \| \| Experiment type \| Expression profiling by array \| \| Summary \| Intestinal epithelia are protected by a layer of mucin secreted by goblet cells against mechanical and chemical injuries, potent causes of inflammation, and the most abundant secreted intestinal mucin is encoded by the Muc2 gene. Genetic deletion of Muc2 causes intestinal inflammation in early stage and tumors after 3 months. The underlying mechanisms are not clear, but epigenetic alterations, particularly, up- and down-regulated microRNAs are involved in the malignant transformation from colitis to cancer. We used miRNA array to profile the differential expression of the miRNAs in Muc2-/- mouse colonic epithelial lin comparison with those in wild-type mice. \| \|  \|  \| \| Overall design \| Total RNA were extracted from mouse colonic epithelial cells and Muc2-/- and +/+, and the RNA were hybridized on Affymetrix miRNA microarray to determine the alterations of miRNAs during colitis development and its malignant transformation from colitis to cancer. To the end, we found miRNA were differential expressed in the Muc2-/- mice, among them 20 miRNAs were significantly downregulated and 71 miRNAs were significantly upregulated in Muc2-/- mice, in comparison with Muc2+/+ mice (change fold >2 or <0.5; T<0.01, p value< 0.05, q value< 0.05). \| \|  \|  \| \| Contributor(s) \| [Bao Y](https://www.ncbi.nlm.nih.gov/pubmed/?term=Bao%20Y%5bAuthor%5d), [Yang W](https://www.ncbi.nlm.nih.gov/pubmed/?term=Yang%20W%5bAuthor%5d) \| \| Citation(s) \| - Bao Y, Guo Y, Li Z, Fang W et al. MicroRNA profiling in Muc2 knockout mice of colitis-associated cancer model reveals epigenetic alterations during chronic colitis malignant transformation. PLoS One 2014;9(6):e99132. PMID: [24941171](https://www.ncbi.nlm.nih.gov/pubmed/24941171) - Bao Y, Chen Z, Guo Y, Feng Y et al. Tumor suppressor microRNA-27a in colorectal carcinogenesis and progression by targeting SGPP1 and Smad2. PLoS One 2014;9(8):e105991. PMID: [25166914](https://www.ncbi.nlm.nih.gov/pubmed/25166914) \| \| Submission date \| Apr 07, 2014 \| \| Last update date \| Jul 27, 2018 \| \| Contact name \| Wancai Yang \| \| E-mail(s) \| [wyang06@uic.edu](mailto:wyang06@uic.edu) \| \| Phone \| 3123554154 \| \| Organization name \| University of Illinois at Chicago \| \| Department \| Pathology \| \| Street address \| 840 South Wood Street \| \| City \| Chicago \| \| State/province \| IL \| \| ZIP/Postal code \| 60612 \| \| Country \| USA \| \|  \|  \| \| Platforms (1) \| \| [GPL16384](https://www.ncbi.nlm.nih.gov/geo/query/acc.cgi?acc=GPL16384) \| [miRNA-3] Affymetrix Multispecies miRNA-3 Array \| \| --- \| --- \| \| \| Samples (8)  [[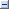](javascript:HandleVisibilityChangeL540101967()) Less...](javascript:HandleVisibilityChangeL540101967()) \| \| [GSM1364101](https://www.ncbi.nlm.nih.gov/geo/query/acc.cgi?acc=GSM1364101) \| Colonic epithelia wild-type mouse 1 \| \| --- \| --- \| \| [GSM1364102](https://www.ncbi.nlm.nih.gov/geo/query/acc.cgi?acc=GSM1364102) \| Colonic epithelia wild-type mouse 2 \| \| [GSM1364103](https://www.ncbi.nlm.nih.gov/geo/query/acc.cgi?acc=GSM1364103) \| Colonic epithelia wild-type mouse 3 \|  \| [GSM1364104](https://www.ncbi.nlm.nih.gov/geo/query/acc.cgi?acc=GSM1364104) \| Colonic epithelia wild-type mouse 4 \| \| --- \| --- \| \| [GSM1364105](https://www.ncbi.nlm.nih.gov/geo/query/acc.cgi?acc=GSM1364105) \| Colonic epithelia Muc2-/- mouse 1 \| \| [GSM1364106](https://www.ncbi.nlm.nih.gov/geo/query/acc.cgi?acc=GSM1364106) \| Colonic epithelia Muc2-/- mouse 2 \| \| [GSM1364107](https://www.ncbi.nlm.nih.gov/geo/query/acc.cgi?acc=GSM1364107) \| Colonic epithelia Muc2-/- mouse 3 \| \| [GSM1364108](https://www.ncbi.nlm.nih.gov/geo/query/acc.cgi?acc=GSM1364108) \| Colonic epithelia Muc2-/- mouse 4 \| \| |
| --- | --- | --- | --- | --- | --- | --- | --- | --- | --- | --- | --- | --- | --- | --- | --- | --- | --- | --- | --- | --- | --- | --- | --- | --- | --- | --- | --- | --- | --- | --- | --- | --- | --- | --- | --- | --- | --- | --- | --- | --- | --- | --- | --- | --- | --- | --- | --- | --- | --- | --- | --- | --- | --- | --- | --- | --- | --- | --- | --- | --- | --- | --- | --- | --- | --- | --- | --- | --- | --- | --- | --- | --- | --- | --- | --- |
