# Supplementary material for: Expression of Concern: Tumor Suppressor MicroRNA-27a in Colorectal Carcinogenesis and Progression by Targeting SGPP1 and Smad2
Source: PLoS One. 2023 Jan 26;18(1):e0280980. doi: 10.1371/journal.pone.0280980 (PMC9879486; doi:10.1371/journal.pone.0280980)

More images of immunohistochemical staining for SGPP1, Smad2 and p-STAT3 in colorectal cancers and normal mucosa (Taken on 12/20/2013)

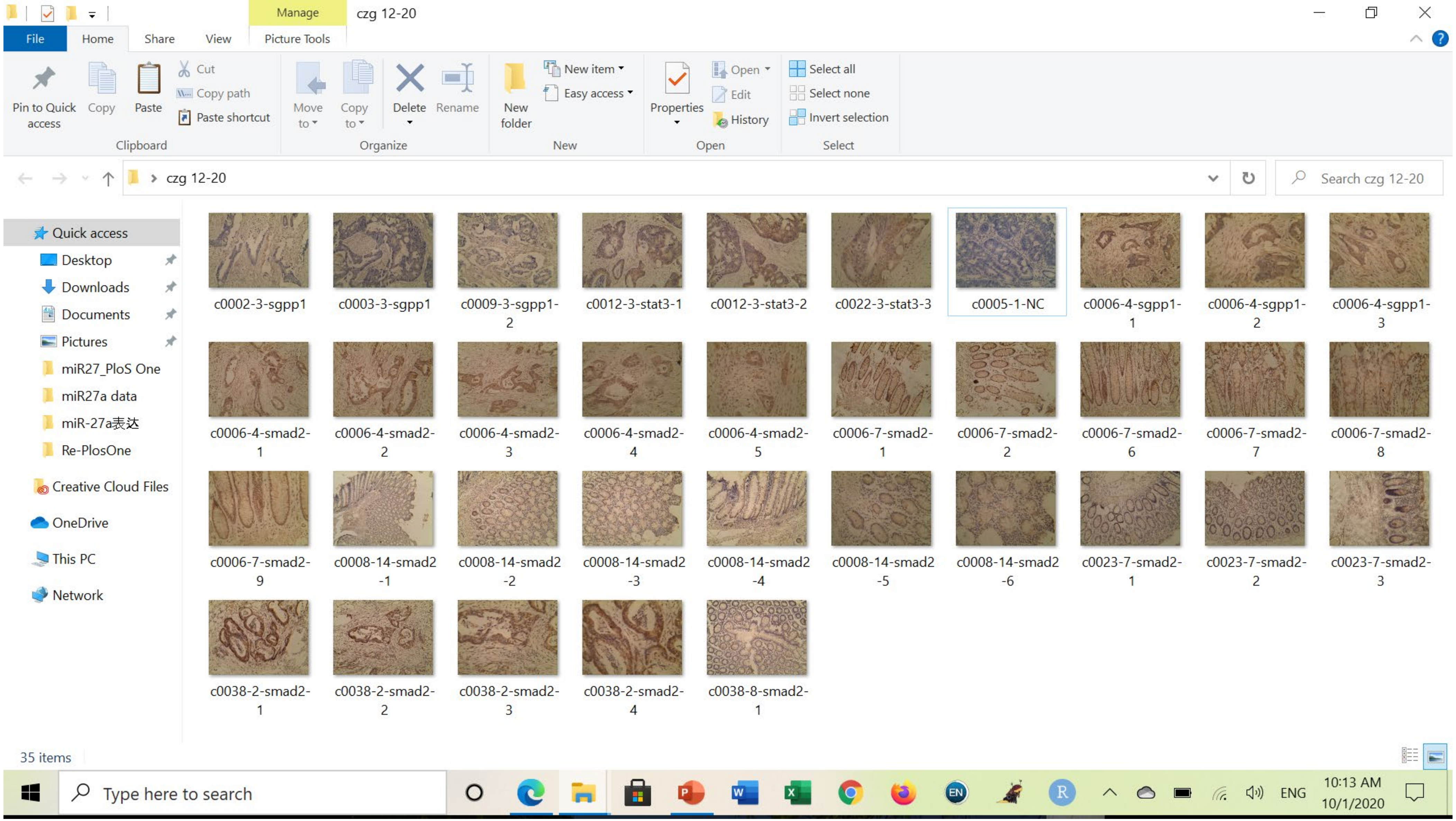

Supplement: S3 File — (ZIP) [file pone.0280980.s003.zip › S3 File - Available underlying data Figure 3/Fig.3B.4F.Images.immunostaining in CRC.pdf]
