# Supplementary material for: Expression of Concern: Tumor Suppressor MicroRNA-27a in Colorectal Carcinogenesis and Progression by Targeting SGPP1 and Smad2
Source: PLoS One. 2023 Jan 26;18(1):e0280980. doi: 10.1371/journal.pone.0280980 (PMC9879486; doi:10.1371/journal.pone.0280980)
Supplement: S4 File — (PDF) [file pone.0280980.s004.pdf]

HCT116, SW480 and Caca0 cells were originally transfected with miR-146a and miR-27a, and the quantifications of transfection with miR-27a in the 3 cell lines were used for the generation of Fig.4A.

Fig.4B

HCT116细胞

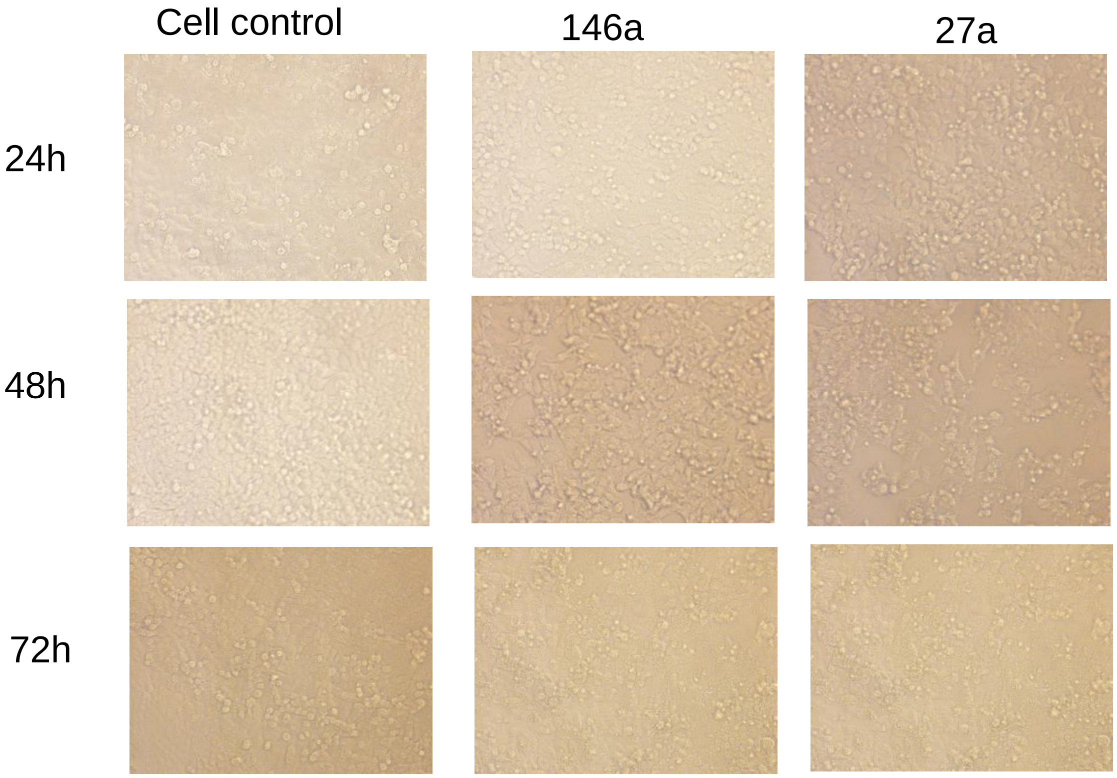

SW480细胞

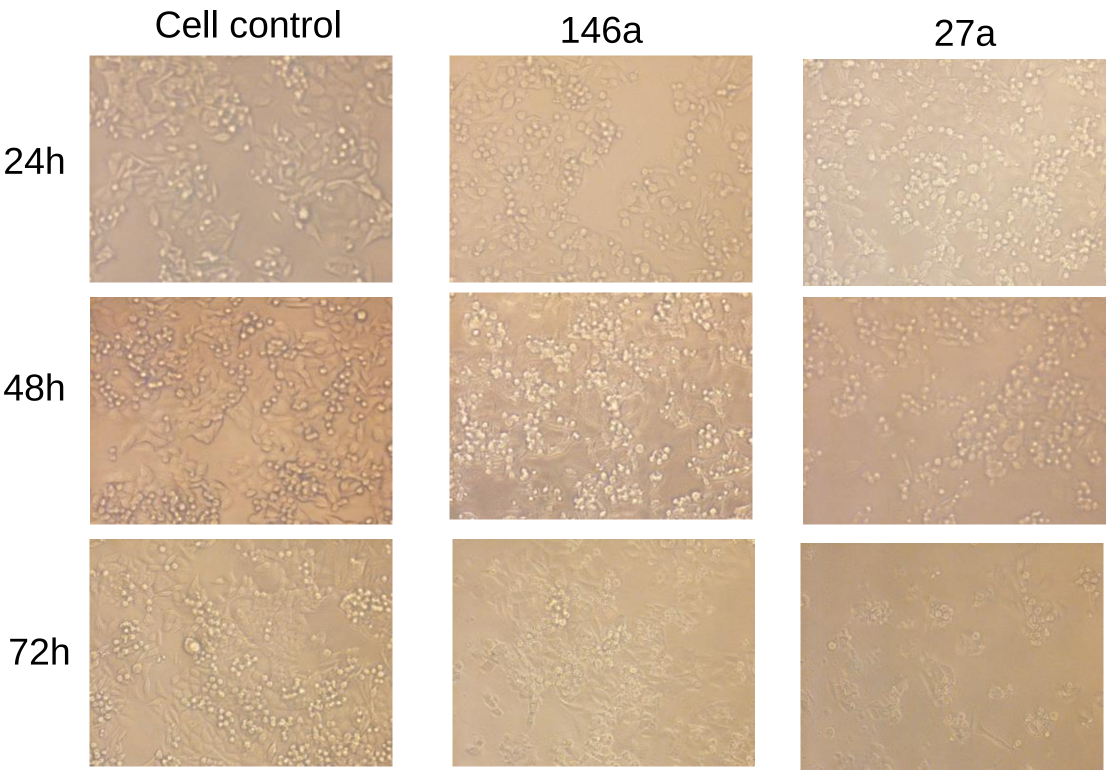

CACO2细胞

Cell control 146a 27a

24h

48h

72h

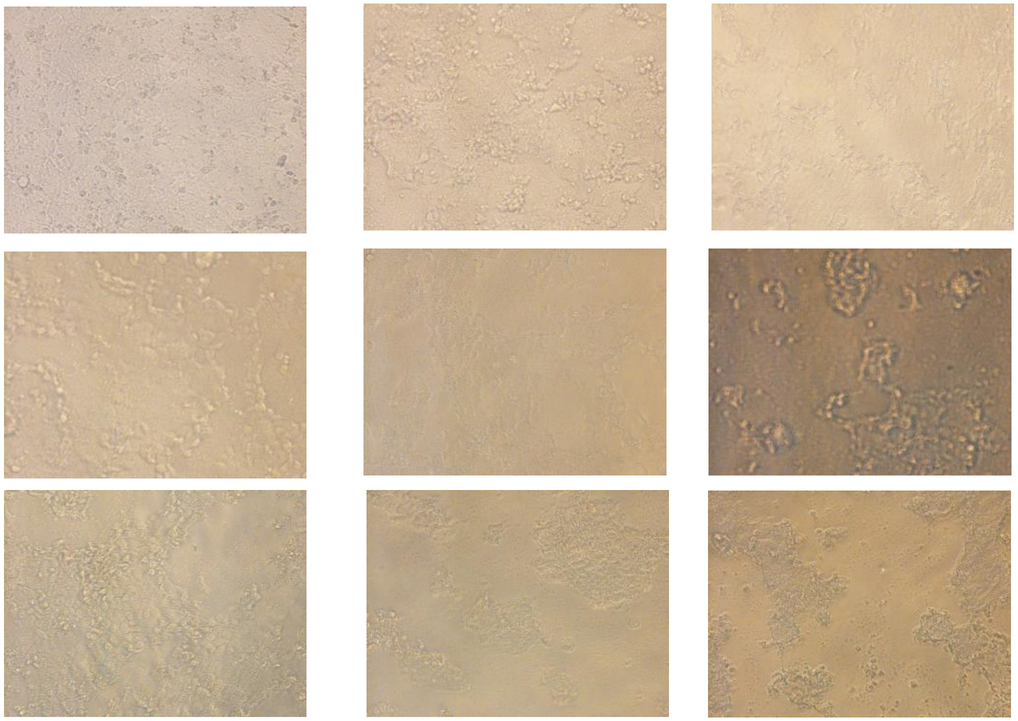

Fig.4C

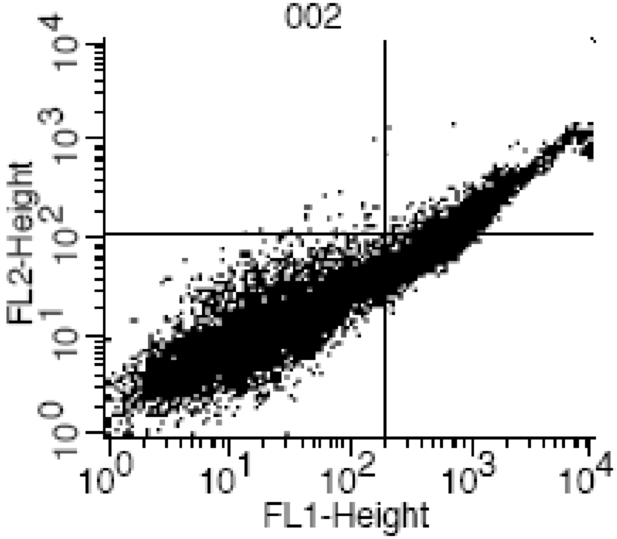

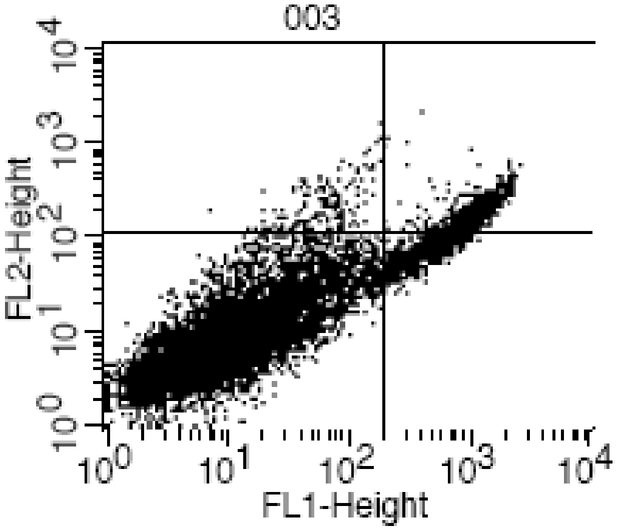

File: 002

Quad	% Gated	% Total
UL	0.35	0.35
UR	22.20	22.20
LL	61.25	61.25
LR	16.20	16.20

File: 003

Quad	% Gated	% Total
UL	1.50	1.50
UR	8.55	8.55
LL	82.38	82.38
LR	7.57	7.57

HCT116 cells transfection with 27a -24h
