# Supplementary figures and images for: Expression of Concern: Tumor Suppressor MicroRNA-27a in Colorectal Carcinogenesis and Progression by Targeting SGPP1 and Smad2
Source: PLoS One. 2023 Jan 26;18(1):e0280980. doi: 10.1371/journal.pone.0280980 (PMC9879486; doi:10.1371/journal.pone.0280980)

## Slide 1
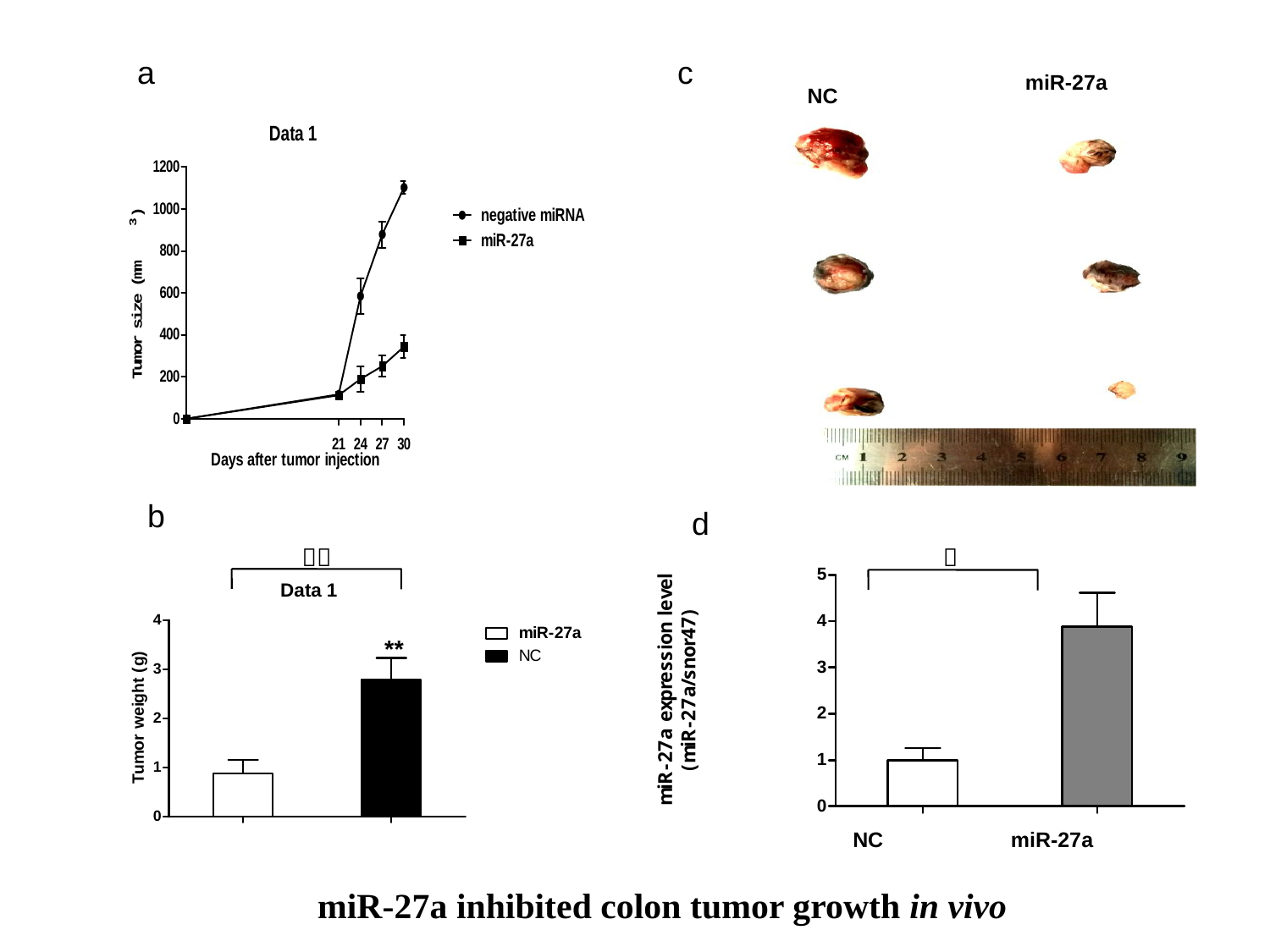

a
c
miR-27a
NC
b
d
＊＊
＊
miR-27a
NC
miR-27a inhibited colon tumor growth in vivo

Supplement: S5 File — (ZIP) [file pone.0280980.s005.zip › S5 File - Available underlying data Figure 5/Fig.5 Xenografts.pptx]
